# Fluorescence-Based Measurements of Membrane-Bound Angiotensin Converting Enzyme 2 Activity Using Xenopus Laevis Oocytes

**DOI:** 10.3390/bios12080601

**Published:** 2022-08-04

**Authors:** Luise Fast, Richard Ågren, Hugo Zeberg

**Affiliations:** 1 Department of Evolutionary Genetics, Max Planck Institute for Evolutionary Anthropology, 04103 Leipzig, Germany; 2 Department of Neuroscience, Karolinska Institutet, 17177 Stockholm, Sweden

**Keywords:** angiotensin converting enzyme 2, microplate reader, Xenopus laevis oocytes, fluorescence intensity, enzymatic assay

## Abstract

Functional investigations of enzymes involving cellular expression systems are important for pharmacological studies. The precise control of expression is challenging in transiently transfected mammalian cell lines. Here, we explored the ability of Xenopus laevis oocytes to express a membrane-bound enzyme for functional characterization using standard 96-well plates and a fluorescence-based plate reader assay. We microinjected oocytes with cRNA encoding the angiotensin converting enzyme 2 (ACE2) and measured the enzymatic activity in single oocytes using a commercial fluorescence-based assay. The injected oocytes showed up to a 50-fold increase in fluorescence compared to uninjected oocytes. This fluorescence intensity was dose-dependent on the amount of *ACE2* cRNA. These results suggest that Xenopus oocytes can be used for the functional evaluation of membrane-bound enzymes, decreasing the experimental workload.

## 1. Introduction

The experimental characterization of enzymatic activity may be performed using various methodologies, including spectrophotometry, calorimetry, chemiluminescence, radiometry, and chromatography [1,2,3]. The enzymes of interest can be purified from native tissues or expressed, e.g., in mammalian cell lines [4,5]. In vitro expression in mammalian cells is typically performed by transient transfection methods, such as lipofection or electroporation. These methods result in variable transfection efficiencies and require several pre-experimental steps [5,6].

In the present investigation, we evaluated Xenopus laevis oocytes as a heterologous enzyme expression system. Xenopus laevis oocytes have traditionally been used for the expression of electrogenic proteins, e.g., ion channels, which may be functionally studied using a two-electrode voltage clamp [7,8,9]. Compared to the transient transfection of mammalian cells, the oocyte expression system allows for the precise stoichiometric control of cRNA amounts and ratios, as oocytes are individually microinjected with cRNA [9]. Oocytes from Xenopus laevis have a full machinery for processing proteins, involving folding, post-translational modifications, and localization to the correct subcellular compartments. Previous studies indicate that Xenopus laevis oocytes display variable autofluorescence [10], potentially complicating the analysis of fluorescent intracellular assays. However, to our knowledge, the utility of Xenopus laevis oocytes in fluorescence-based assays remains largely unexplored.

Angiotensin converting enzyme 2 (ACE2) is the main extracellular target of the SARS-CoV-2 spike protein, which mediates the initial step in the cellular infection [11]. The enzymatic reaction of ACE2 involves the degradation of the peptide angiotensin II into a smaller peptide consisting of the seven first N-terminal amino acids, Ang-(1–7) [12,13]. Here, we use a fluorescence-based assay developed for lysed cells or tissues to investigate whether oocyte-expressed ACE2 degrades its substrate extracellularly.

## 2. Materials and Methods

### 2.1. Molecular Biology

**Figure 1:**
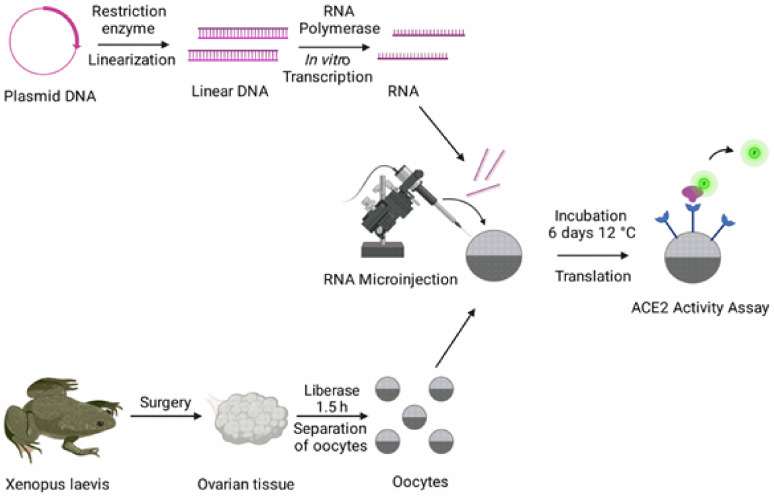
**Overview of the methodology for fluorometric ACE2 activity measurements in Xenopus laevis oocytes.** See Methods for detailed steps. The image was created with Biorender.com (accessed on 10 August 2021).

Human *ACE2* (GenScript, Piscataway, NJ, USA) was cloned into the plasmid vector Xenopus oocyte or mammalian (pXOOM) [14]. The Xanthomonas badrii restriction enzyme (XbaI) was used to linearize the *ACE2* plasmid in the presence of Buffer H (SuRE/Cut™, Roche, Switzerland). The linearized *ACE2* was purified using the PureLink™ PCR Purification Kit (Thermo Fisher Scientific, Waltham, MA, USA). The purified *ACE2* was transcribed in vitro using the T7 mMessage mMachine kit (Ambion, Austin, TX, USA; see Figure 1 for an overview of the procedures). The linearization and in vitro transcription were performed at 37 °C. The RNA concentration and purity were determined using the Nanodrop 1000 spectrophotometer (Thermo Fisher Scientific).

### 2.2. Oocyte Preparation

Oocytes from the African clawed toad, Xenopus laevis, were surgically isolated as described previously [15]. The procedure conforms to Directive 2010/63/EU and has been approved by the Swedish National Board for Laboratory Animals and the Animal Welfare Ethical Committee in Stockholm (approval number N686/21). All experiments were performed in accordance with relevant guidelines and regulations. Following 24 h of incubation at 12 °C, oocytes were microinjected with 0.02–20.54 ng *ACE2* cRNA using the Nanoject III (Drummond Scientific, Broomall, PA, USA) and a volume of 50 nL per oocyte (see Figure 1). The oocytes were then incubated for 6 days at 12 °C in atmospheric air.

### 2.3. ACE2 Activity Assay

The ACE2 Activity Assay was performed using the fluorometric Angiotensin II Converting Enzyme Activity Assay Kit (Abcam, Cambridge, UK; Cat. Ab273297). For the oocytes, 50 µL ACE2 Assay Buffer were added per well of a black-wall 96 well plate (Greiner Bio-One, Kremsmünster, Austria; Cat. 655090) in which one oocyte per well was transferred. After 15 min incubation at room temperature, 50 µL ACE2 substrate (diluted 1:25 in ACE2 Assay Buffer) were added per well. Fluorescence was measured in a kinetic mode (excitation and emission wavelengths at 320 and 420 nm, respectively) during 130 cycles (30 s cycle time), using the CLARIOstar^®^ microplate reader (BMG Labtech, Ortenberg, Germany). The experiments were performed at room temperature (22 °C).

### 2.4. Data Analysis and Statistics

Fluorescence data were analyzed with the CLARIOstar^®^ MARS software (BMG Labtech, Ortenberg, Germany). The slopes of all kinetic curves were calculated by fitting a straight line between the time points of 15 and 30 min of the enzymatic reaction. Statistical analysis was performed using Prism 6.0 (GraphPad, San Diego, CA, USA). A one-way analysis of variance (ANOVA) with Dunnett’s multiple comparisons test and *t*-tests were used for statistical comparison. *p* < 0.05 was considered statistically significant.

## 3. Results

**Figure 2:**
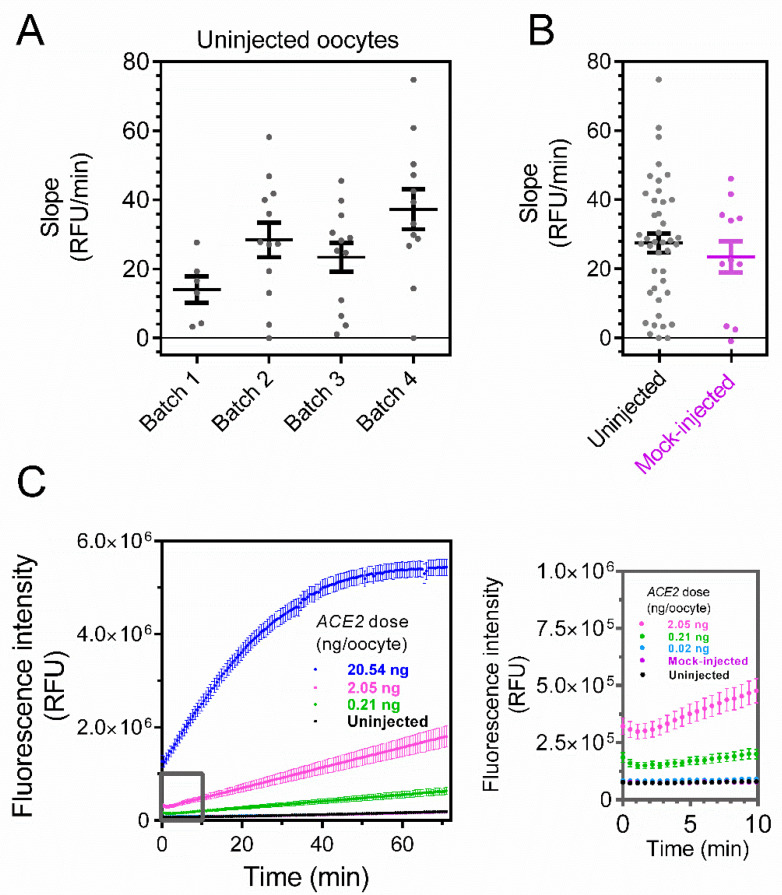

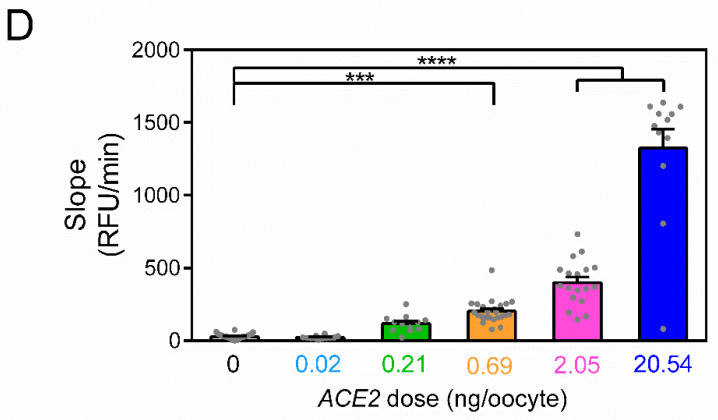
**ACE2 activity in Xenopus laevis oocytes is dose-dependent on the amount of injected cRNA encoding the enzyme**. (**A**) Fluorometric ACE2 activity (measured as slope between 15 and 30 min) in four batches of uninjected oocytes. Each dot represents a biological replicate (N = 6, 12, 12 and 12 oocytes per batch, respectively for batches 1–4). (**B**) Fluorometric ACE2 activity for all uninjected oocytes in (**A**) (slope = 27.4 ± 2.7 RFU/min, N = 42) and mock-injected oocytes (slope = 23.4 ± 4.5 RFU/min, N = 12). ACE2 activity does not differ between uninjected and mock-injected oocytes (*p* = 0.48, unpaired *t*-test). (**C**) Representative traces of ACE2 activity in uninjected, mock-injected, and *ACE2* cRNA-injected oocytes (0.02–20.54 ng of cRNA). Inset (grey) shows the initial 10 min. (**D**) ACE2 activity in uninjected and injected oocytes (0.02–20.54 ng *ACE2* cRNA). The number of oocytes was 42 for uninjected, 12 for 0.02, 0.21, and 20.54 ng, 25 for 0.69 ng, and 18 for 2.05 ng *ACE2* cRNA. ***, *p* < 0.001 and ****, *p* < 0.0001 for ANOVA with Dunnett’s multiple comparisons test. Horizontal bars indicate average values and error bars represent standard errors of the mean.

As a first step in evaluating the performance of the oocyte expression system and its signal-to-noise ratio, we used the ACE2 assay for uninjected oocytes derived from four batches. Single oocytes were placed in a 96-well plate, and the fluorometric activity was recorded every 30 s per well. Over a period of 70 min, stable baselines were observed for all batches (Figure 2A). To rule out that the injection itself influences the baseline (e.g., by damaging the oocyte membrane and causing leakage), we investigated the ACE2 activity using oocytes mock-injected with nuclease-free water. Mock-injected oocytes showed a slope of 23.4 relative fluorescence units per min (RFU/min; standard error of the mean [SEM] = 4.5 RFU/min, *n* = 12) that did not differ from the slopes of uninjected cells (27.4 RFU/min, SEM = 2.7 RFU/min, *n* = 42; *p* = 0.48, unpaired *t*-test; Figure 2B).

Having established the background conditions, we injected oocytes with increasing amounts of cRNA encoding ACE2. Lower amounts of *ACE2* cRNA (0.02 and 0.21 ng) did not evoke a significant increase in the signal compared to the uninjected or mock-injected oocytes (ANOVA; Figure 2C,D; see inset). For larger amounts of cRNA, a significant increase in the signal is observed in the timespan of 15–30 min. The signal increased in a dose-dependent manner, reaching 1324 RFU/min (SEM = 132 RFU/min, *n* = 12) for oocytes injected with 20.54 ng of *ACE2* cRNA (Figure 2D).

## 4. Discussion

We used Xenopus laevis oocytes as an enzyme expression system. The enzymatic activity in single oocytes was characterized using methods similar to fluorescent recordings of transfected mammalian cells. Compared to the expression when using transfection plasmids, which show great variability in transfection efficiency, the direct injection of cRNA reduces cell-to-cell variability (Figure 2D).

Moreover, with each oocyte considered as a separate replicate, the use of standard 96-well plates allows for a large number of biological replicates in one run. The most time-consuming part of the protocol is the injection of cRNA into oocytes. However, with an established pipeline, an oocyte can be injected using a microinjector in ~10 s or less, and automated injection methods are available [16].

For any kinetic assay, the signal-to-noise ratio impacts the resolution. Here, both uninjected and mock-injected oocytes show virtually no signal when compared to injected oocytes, as demonstrated in Figure 2D. Thus, the proposed protocol allows for both a high throughput and high resolution. Xenopus laevis oocytes may therefore be used as a quick, simple, and affordable system to express ACE2 and study how ligands affect ACE2 activity.

ACE2 is the main receptor for the SARS-CoV-2 virus and mediates the initial step in cellular infection [11]. The analysis of ligands modulating the interaction between the spike protein of SARS-CoV-2 and ACE2 may thus help to further understand the entry of the virus into its host cells [17]. Xenopus laevis oocytes may be used as a swift, simple, and affordable system to express ACE2, which may be used to study how ligands affect ACE2 activity. For example, peptides mimicking motifs in the spike-protein of SARS-CoV-2 (and sequence variation thereof) could be tested in terms of their ability to affect ACE2 activity.

As shown here and by others, Xenopus laevis oocytes efficiently express many mammalian membrane-bound proteins [18]. However, in any particular case, glycosylation and other posttranslational modifications may differ between Xenopus laevis oocytes and mammalian cells. In each particular case, this may therefore need to be investigated in order to exclude differences impacting results.

In summary, Xenopus laevis oocytes express ACE2 in a dose-dependent manner, as measured by enzymatic activity using fluorescence intensity. Xenopus laevis oocytes are thus a valuable tool for studying membrane-bound enzymes.

## Data Availability

All data generated or analyzed during this study are included in this published article.
